# Cortisol responses enhance negative valence perception for ambiguous facial expressions

**DOI:** 10.1038/s41598-017-14846-3

**Published:** 2017-11-08

**Authors:** Catherine C. Brown, Candace M. Raio, Maital Neta

**Affiliations:** 1 Department of Psychology, University of Nebraska-Lincoln, 238 Burnett Hall, Lincoln, NE 68588 USA; 2 Center for Neural Science, New York University, 4 Washington Place, Room 809, New York, NY 10003 USA

## Abstract

Stress exposure elicits a prolonged neuroendocrine response, marked by cortisol release, which can influence important forms of affective decision-making. Identifying how stress reactivity shapes subjective biases in decisions about emotional ambiguity (i.e., valence bias) provides insight into the role stress plays in basic affective processing for healthy and clinical populations alike. Here, we sought to examine how stress reactivity affects valence decisions about emotional ambiguity. Given that stress prioritizes automatic emotional processing which, in the context of valence bias, is associated with increased negativity, we tested how individual differences in acute stress responses influence valence bias and how this decision process evolves over time. Participants provided baseline ratings of clear (happy, angry) and ambiguous (surprised) facial expressions, then re-rated similar stimuli after undergoing an acute stress or control manipulation a week later; salivary cortisol was measured throughout to assay stress reactivity. Elevations in cortisol were associated with more negative ratings of surprised faces, and with more direct response trajectories toward negative ratings (i.e., less response competition). These effects were selectively driven by the stress group, evidencing that increased stress reactivity is associated with a stronger negativity bias during ambiguous affective decision-making.

## Introduction

Daily life is marked by exposure to stressors known to elicit neurophysiological responses that exert powerful effects on brain function and behavior. Recent estimates suggest that over 75% of Americans report experiencing recent stress that has had deleterious effects on interpersonal, professional, health-related, and financial decisions^1^. Stressors rapidly induce sympathetic nervous system arousal resulting in noradrenergic activation, followed by the recruitment of the Hypothalamic-Pituitary-Adrenal axis (HPA-axis), which triggers the systemic release of glucocorticoids (i.e., cortisol)^2^. Research points to the inherent uncertainty surrounding a stressor as a seminal driver of these neurophysiological responses^3,4^. The tightly coupled relationship between stress and uncertainty raises the important question of how stress exposure affects the way individuals behave when encountering ambiguity in their environment. While a large body of research has examined this question in the context of economic decision-making, far less work has examined the effects of stress on decisions about ambiguous affective stimuli encountered in daily life.

Biologically relevant cues, such as facial expressions, offer an ecologically valid and highly pertinent model for examining such affective decision-making. Facial expressions not only convey the affective state of a social agent, but also provide important predictive information about one’s environment such as the presence of potential rewards or threats, which can inform motivational significance and drive subsequent affective and behavioral responses. While some facial expressions provide clear, unequivocal information about the valence of another’s affective state (e.g., happiness, anger), others—such as surprised expressions—are ambiguous since they can signal the presence of positive *or* negative events. The ambiguous nature of surprised facial expressions affords researchers the opportunity to identify subjective biases that individuals may have when determining the valence of these faces.

Since valence bias for ambiguous facial expressions varies widely across individuals, it therefore provides a quantifiable index of one’s propensity to interpret the valence of environmental ambiguity^5–8^. Despite individual differences in valence bias, research now points to negativity as being the initial, automatic response to surprised expressions^8–10^. This predisposed tendency for negativity is marked by higher amygdala activity and faster reaction times^5,10^, whereas positive ratings emerge after longer reaction times and the recruitment of prefrontal networks^5,8–11^. These findings support a provisional model for the appraisal of surprised faces as being predisposed to be negative, with additional processing or regulatory influence required to shift the predictive value of such cues from negative and to positive.

A growing body of work points to stress reactivity as an important factor that can modulate the appraisals of ambiguous facial expressions. Negative appraisals of ambiguous faces are associated with increased trait anxiety^12^, depression^13^, and higher amygdala responses^5,10^. Additionally, acute stress exposure has been linked to negative affect^4^, diminished cognitive control and flexibility^14–16^, and reduced regulation of negative emotion^17^ and threat responses^18^. Elevated stress hormones such as catecholamines (e.g. dopamine, noradrenaline) and glucocorticoids (i.e., cortisol) reduce prefrontal control and enhance amygdala activity that supports the detection of biologically salient cues^19–21^. Finally, acute stress has been proposed to shift the allocation of neural processing away from controlled, executive function networks and toward those that facilitate the detection of threats^21^. This work collectively points to the hypothesis that higher stress reactivity might bias individuals to appraise ambiguous stimuli more negatively and further diminish regulatory capacity that may otherwise attenuate this negative bias during affective decision-making. Furthermore, as individuals recover from acute stress and regulatory capacity returns, negative appraisals of ambiguity may diminish to pre-stressor levels. To this end, we directly tested the effect of stress on valence bias of ambiguous facial expression by causally manipulating acute stress levels in healthy individuals. Our primary goal was to assess whether stress reactivity affects the perceived valence of ambiguous social cues and track the dynamics of the choice process prior to the final valence decision.

## Methods

### Participants

An a priori power analysis using prior research^22^ indicated a requisite sample size of 21 participants per group to replicate a large effect size (*d* = −0.89) with 80% power and α = 0.05, when comparing group cortisol levels 10 minutes post-stressor or post-control. Fifty-two participants were recruited from the University of Nebraska-Lincoln. Eligible participants were right-handed and reported having no history of psychiatric or neurological disorders. Three participants were excluded for either failing to return for the second session, providing insufficient quantities of saliva, or poor behavioral performance (i.e., rating happy expressions as negative and angry expressions as positive). Four additional participants were removed for having cortisol change greater than two standard deviations above or below the group mean. The final sample included 22 participants randomly assigned to the stress group (11 female, mean age = 20.36, *SD* = 2.15) and 23 participants randomly assigned to the control group (11 female, mean age = 20.04, *SD* = 3.57). Due to a programming error, two participants’ responses to the individual difference questionnaire were not recorded. All participants received course credit or monetary compensation after completing each session. Written informed consent was obtained from participants before each session, and the University of Nebraska Committee for the Protection of Human Subjects approved all procedures. All methods were performed in accordance with the relevant guidelines and regulations of the Committee.

### Face Stimuli

Stimuli were drawn from a previously compiled set of 48 images of faces, 24 with an ambiguous valence (surprise), and 24 with a clear valence (12 angry and 12 happy)^7^. Thirty-four discrete identities (17 male) were included, though not all identities were represented in each expression. Fourteen identities (7 male) came from the NimStim standardized facial expression stimulus set^23^. Development of this stimulus set was overseen by Nim Tottenham and supported by the John D. and Catherine T. MacArthur Foundation Research Network on Early Experience and Brain Development. Twenty additional identities (10 male) came from the averaged Karolinska Directed Emotional Faces database^24^.

### Procedure

**Figure 1:**
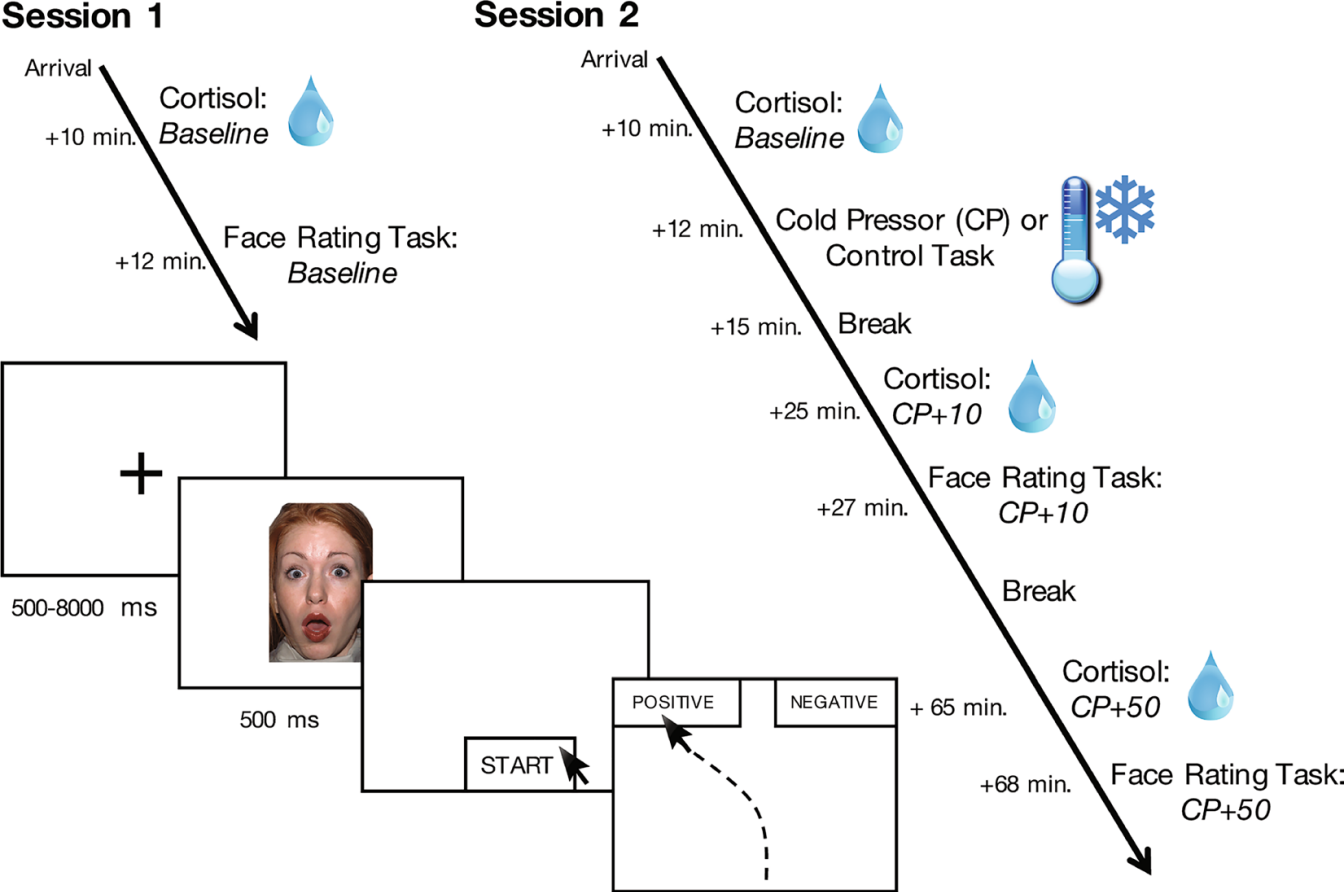
Timeline of experimental procedure and cortisol assessments. The facial expression depicted here is part of the NimStim standardized facial expression stimulus set^23^ and is used with permission. Development of this stimulus set was overseen by Nim Tottenham and supported by the John D. and Catherine T. MacArthur Foundation Research Network on Early Experience and Brain Development. Please contact Nim Tottenham at tott0006@tc.umn.edu for more information concerning the stimulus set. All other graphics are within the public domain under CC0 license.

Participants completed two sessions a week apart, with the face-rating task occurring at three distinct time points (see Fig. 1 for experimental timeline). On Day 1, participants gave informed consent before completing the face-rating task in order to provide a baseline valence-rating measure. One week later, participants returned and were again consented before being randomly assigned to either the stress or control group (see *Stress manipulation and measurement* below for full procedure). Participants repeated the face-rating task ten minutes after the stress or control task, when cortisol levels in the stress group were expected to rise, and again one final time 50 minutes later when cortisol levels were expected to return to baseline. Saliva samples were collected throughout to assay fluctuations in cortisol concentrations.

#### Face-rating task

Each iteration of the task included 16 faces (8 surprised, 4 happy, and 4 angry), each presented four times in randomized order, for a total of 64 trials. A different set of 16 faces was presented for both subsequent rating tasks. During each trial, a black fixation cross appeared in the center of a white background for 500–8000 ms preceding a 500 ms face presentation, after which participants indicated whether they thought the expression was positive or negative by clicking a start button at the bottom of the display, then clicking one of the two response option buttons in the upper left- or upper right-hand corner of the display. The fixation timing was randomized in 500-ms intervals. Each iteration of the rating task lasted approximately 5 minutes.

#### Mouse-tracking assessment

MouseTracker software was used to present the stimuli and to record the mouse trajectories of each response^25^. MouseTracker software provides a more sensitive measure of negativity beyond explicit valence ratings by tracking the trajectory of mouse movements as participants determine the valence of ambiguous facial expressions. During a given trial, a participant’s mouse can either move directly from the start button to their response (i.e., in a straight line) or they may be pulled to some degree toward the opposite response during the decision process (i.e., portraying curvature in their response trajectory). Thus, the mouse’s trajectory reflects the implicit competition between positivity and negativity ratings on each trial.

#### Stress manipulation and measurement

To control for diurnal rhythms in stress hormone levels, participants were run between 12:00 pm and 5:00 pm and at the same time of day for each session. At the start of each experimental session, participants acclimated to the laboratory setting for 10 minutes before providing a baseline saliva sample to assay resting cortisol concentrations. On Day 2, based on the procedures used by Raio and colleagues (2013)^22^, participants then either underwent the cold-pressor task^26^, which required the continuous submersion of the right forearm in an ice-water bath (0–4 °C, stress group), or a matched control task using warm water (~37 °C, control group), for three consecutive minutes. Participants provided additional saliva samples 10 minutes after the stress or control task to allow sufficient time for cortisol responses to begin to rise. This timeline enabled us to measure the face-rating task while the stress response was beginning to peak, thus capturing stress-related change from both cortisol and the earlier noradrenergic responses, which are known to work synergistically with cortisol to affect brain function and behavior^19–21^. Indeed, this timeline is consistent with past studies investigating the effects of acute stress exposure on different forms of affective and cognitive processes^15,22,27,28^. A final saliva sample was collected 50 minutes later in order to assess the recovery of stress responses. During this time, participants were provided neutral reading material, crosswords, and puzzles, and were instructed to stay awake. As a subjective measure of stress, participants reported how stressful they found the task on a scale of 1–9, with 1 being the least stressful and 9 being the most stressful, immediately following the cold-pressor or control task.

#### Neuroendocrine assessment

Saliva was collected using SalivaBio Oral Swabs (Salimetrics LLC, Philadelphia, PA). At each collection time point, one swab was placed under the tongue for 2 minutes. Samples were kept on ice during each session and were then immediately frozen at −20 °C, where they remained stored until analysis. Samples were analyzed at the University of Nebraska-Lincoln Salivary Bioscience Laboratory using a commercially-available competitive enzyme immunoassay kit for salivary cortisol (Salimetrics LLC, Philadelphia, PA). Intra- and inter-assay coefficients of variation were less than 6%.

#### Individual Difference Questionnaires

To measure individual differences in emotion regulatory ability, participants also completed the Difficulties in Emotion Regulation Scale (DERS)^29^. This 36-item questionnaire assesses aspects of emotion dysregulation and provides a total score as well as six sub-scores: non-acceptance of emotional responses, difficulties engaging in goal directed behavior, impulse control difficulties, lack of emotional awareness, limited access to emotion regulation strategies, and lack of emotional clarity.

### Statistical Analysis

Analysis of variance (ANOVA) with repeated measures was used to analyze all face ratings and neuroendocrine data. Post hoc comparisons were conducted using Student *t*-tests when appropriate. All tests were two-tailed and considered statistically significant when *p* < 0.05. Correlational analyses used Spearman correlations if tests of normality (Shapiro-Wilk) indicated that values were not normally distributed. All analyses were conducted using SPSS (version 20.0, 2011; IBM Corp., Armonk, NY) and RStudio (version 1.0.136; RStudio, Inc., Boston, MA). The datasets generated during and/or analyzed during the current study are available from the corresponding author on reasonable request.

## Results

### Stress Reactivity Measures

**Figure 2:**
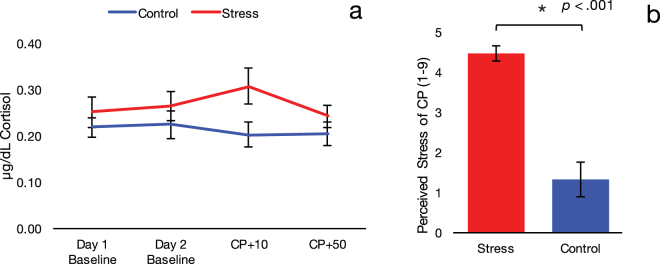
(**a**) Cortisol at 10 minutes post-stressor (CP+ 10) was significantly higher for the stress group than control group. (**b**) Perceived stress at ten minutes post-stressor was significantly higher in the group that underwent the cold-pressor task relative to controls (*p* < 0.001). Error bars indicate SE.

On Day 1, as expected given that we did not manipulate stress levels, baseline cortisol levels did not differ between groups (*t*_(43)_ = −0.86, *p* = 0.38). On Day 2, to verify the efficacy of our stress induction technique, we conducted a Group (stress, control) × Time (baseline, 10 minutes post-stressor, 50 minutes post-stressor) repeated-measures ANOVA. Critically, this analysis revealed a significant Group × Time interaction, *F*(2, 86) = 3.61, *p* = 0.031, partial η^2^ = 0.08. Bonferroni-corrected post-hoc comparisons indicated that cortisol at 10 minutes post-stressor (CP+ 10) was significantly higher for the stress group (*M* = 0.31, 95% CI [0.24, 0.38]) than the control group (*M* = 0.20, [0.24, 0.27]), *p* = 0.033. Additionally, cortisol concentrations in the stress group at CP+ 10 were significantly greater relative to baseline (*M* = 0.26, [0.20, 0.33], *p* = 0.046) and to 50 minutes post-stressor (CP+ 50) (*M* = 0.24, [0.19, 0.29], *p* = 0.012). Consistent with this finding, perceived stress was also significantly higher in the group that underwent the cold-pressor task relative to controls (*t*_(43)_ = 6.84, *p* < 0.001), confirming that both subjective and neuroendocrine stress measures were selectively elevated in the stress group only on Day 2 (Fig. 2). As reported in past research^22^, perceived stress ratings after the stress/control task (on a scale of 1 to 9) were correlated with increases in cortisol relative to baseline, *r*_s_ = 0.47, *p* = 0.001. Thus, individuals who reported greater perceived stress showed a more robust neuroendocrine response to the stress task.

### Stress Effects on Valence Ratings

As expected, angry faces were rated as consistently negative (*M* = 98.93%, [98.22, 99.64]) and happy faces were rated as consistently positive (*M* = 1.53%, [0.58, 2.98]) across participants. To assess valence bias when rating ambiguous faces, we examined the percentage of trials in which surprise was rated as negative. A Group (stress, control) x Time (baseline, 10 minutes post-stressor, 50 minutes post-stressor) repeated-measures ANOVA revealed that ratings of surprised faces did not differ between or within groups across any time points (all *p*s > 0.250), indicating that valence bias decisions did not change over time as a function of acute stress exposure (i.e., group assignment).

**Figure 3:**
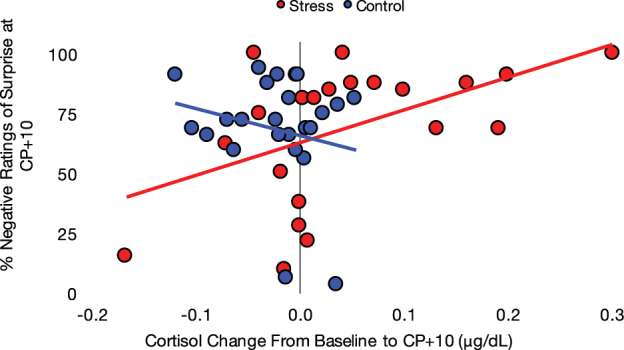
There was a significant positive correlation between the stress group’s percent negative ratings of surprised faces 10 minutes post-stressor and their changes in cortisol from baseline to 10 minutes post-stressor. This correlation was not significant for the control group.

Given the notable effects of cortisol on the neural circuitry underlying affective decision-making, we next examined whether individual differences in stress reactivity were associated with valence ratings on Day 2. Therefore, we examined the relationship between changes in cortisol relative to baseline and ratings of surprised faces after the CP/control task. This analysis revealed a significant positive correlation between the stress group’s ratings at CP+ 10 and their change in cortisol from Day 2 baseline to CP+ 10, *r*(20) = 0.50, *p* = 0.019, such that individuals with a greater neuroendocrine response to the stressor rated surprise more negatively post-stressor. This correlation was not significant in the control group, *r*(21) = −0.22, *p* = 0.322. Further, we confirmed that these two correlations were significantly different from each other, *z* = −2.38, *p* = 0.017 (Fig. 3). To account for extreme scores or outliers, we assessed these correlations using Spearman’s rank-order correlation and found that the effect remained for the stress group, *r*_s_ = 0.47, *p* = 0.029, and not for the control group, *r*_s_ = −0.12, *p* = 0.587. The two rank-order correlations were significantly different from each other, *z* = −1.97, *p* = 0.049.

We next assessed whether the recovery of cortisol responses was related to valence ratings at this same post-stress time point (i.e., CP+ 10). That is, where valence ratings 10 minutes after the stressor related to how well individuals’ cortisol levels subsequently recovered to baseline. Here, we found a trend toward a negative correlation between how well individuals’ cortisol levels subsequently recovered to baseline (i.e., change from 10 to 50 minutes post-stress) and valence ratings after the stressor, *r*_s_ = −0.39, *p* = 0.070. As before, this trend did not emerge for the control group, *r*_s_ = −0.08, *p* = 0.715. Collectively, these results suggest that individual differences in cortisol reactivity and recovery—but not stress exposure per se—relate to negativity ratings in a dissociated manner.

### Stress Effects on Mouse Trajectories

**Figure 4:**
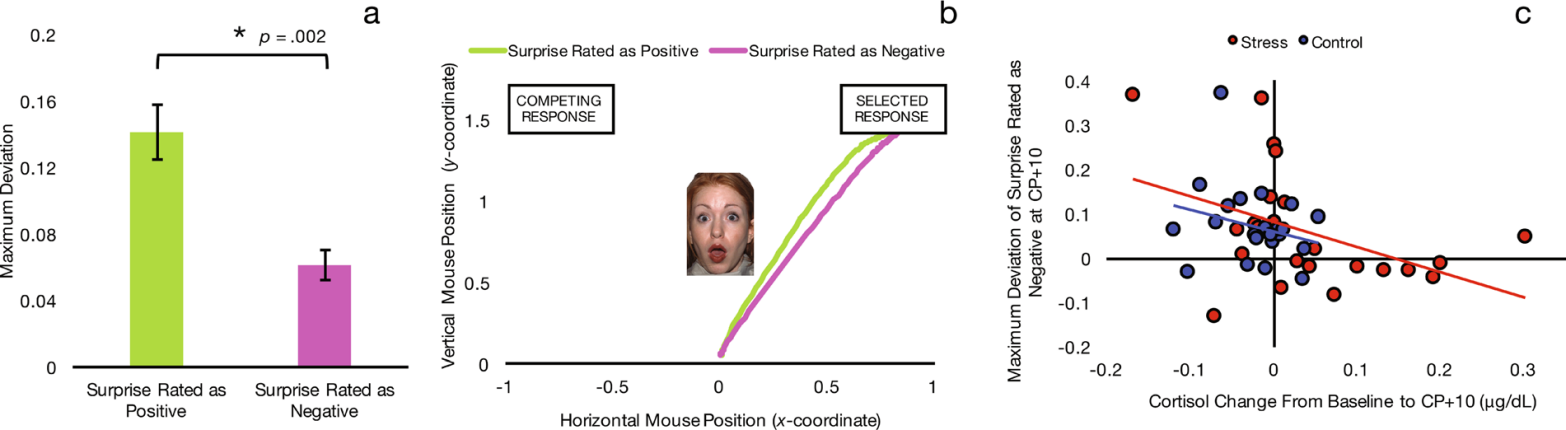
(**a**) Mouse trajectories indicate that participants were more drawn to “negative” when rating surprise as positive than they were drawn to “positive” when rating surprise as negative, confirming prior research suggesting that people have an overall bias towards negativity. Error bars indicate SE. (**b**) Trajectories for surprise rated as positive and surprise rated as negative, averaged across all participants and time points. (**c**) Cortisol change from baseline to post-stressor showed a significant negative relationship with MD of surprise rated as negative following stressor/control. This correlation was significant only in the stress group. The mouse trajectory of those who had greater cortisol increases was more directly negative. The facial expression depicted here is part of the NimStim standardized facial expression stimulus set^23^ and is used with permission. Development of this stimulus set was overseen by Nim Tottenham and supported by the John D. and Catherine T. MacArthur Foundation Research Network on Early Experience and Brain Development. Please contact Nim Tottenham at tott0006@tc.umn.edu for more information concerning the stimulus set.

We calculated an average maximum deviation (MD) for each subject when rating surprise as negative and when rating surprise as positive. MD quantifies the attraction toward the unselected response by measuring the largest perpendicular deviation away from the most direct trajectory to the selected response. A larger MD indicates greater competition of the alternative response. A Group (stress, control) × Valence (surprise rated as positive, surprise rated as negative) × Time (baseline, 10 minutes post-stressor, 50 minutes post-stressor) repeated-measures ANOVA using MD revealed a main effect of Valence, *F*(1, 85) = 11.27, *p* = 0.002, partial η^2^ = 0.20, such that MD of rating surprise as positive was greater than rating it as negative (Fig. 4a). This indicates that participants were more drawn toward the negative response when rating surprise as positive than they were drawn toward the positive response when rating surprise as negative.

MD was also useful in identifying specific effects of the stress manipulation. Specifically, we examined the linear relationships between cortisol change from baseline to post-stressor and MD of surprise rated as negative and positive. While MD of surprise rated as positive showed no relationship with cortisol change, *r*(43) = 0.02, *p* = 0.874, MD of surprise rated as negative at CP+ 10 was negatively related to cortisol change, *r*(43) = −0.38, *p* = 0.010, such that participants with a greater increase in cortisol showed less attraction toward the positive response when rating surprise as negative (i.e., they moved their mouse more directly toward the negative response). This finding suggests that post-stressor negativity was more automatic for those with a greater cortisol increase (Fig. 4b). This correlation was significant for the stress group, *r*(20) = −0.43, *p* = 0.044, but not for the control group, *r*(21) = −0.25, *p* = 0.259. We also examined the relationship between perceived stress post-task and MD of surprise rated as negative and found a negative correlation, *r*(43) = −0.38, *p* = 0.011, such that those who found the CP task more stressful moved their mouse more directly to the negative response and had less competition from the positive response.

### Difficulties in Emotion Regulation Scale (DERS)

Finally, we tested the linear relationship between DERS scores and the proportion of negative ratings for surprised faces post-stressor. Participants’ scores on the DERS subscales were related to both the behavioral ratings and changes in cortisol. First, greater difficulties with goal-directed behavior was associated with more negative ratings of surprise at CP+ 10, *r*(41) = 0.35, *p* = 0.024, and with changes in cortisol from baseline to CP+ 10, *r*(41) = 0.31, *p* = 0.042. The latter correlation was significant for the stress group at trend *r*(18) = 0.44, *p* = 0.052, but not for the control group, *r*(21) = −0.10, *p* = 0.651, with the two correlations being significantly different at trend, *z* = 1.73, *p* = 0.084. These findings show that participants who had more difficulties engaging in goal-directed behavior provided more negative ratings for surprised faces and had higher stress reactivity following the stressor/control manipulation.

Further, more limited access to emotion regulation strategies was also associated with more negative ratings of surprise at CP+ 10, *r*(41) = 0.31, *p* = 0.044, which was significant in the stress group, *r*(18) = 0.49, *p* = 0.029, and not the control group, *r*(21) = 0.15, p = 0.508. Also within the stress group, more limited access to emotion regulation strategies showed a (marginally) significant relationship with greater increases in cortisol from baseline to CP+ 10, *r*(18) = 0.43, *p* = 0.056. This was not true for the control group, and the two correlations were (marginally) significantly different, *z* = 1.92, *p* = 0.055. These findings reveal that stressed participants who could not utilize emotion regulation strategies rated surprise more negatively after the manipulation, and experienced greater cortisol increases after the stressor.

## Discussion

A recent surge of work has identified stress exposure as a prominent factor that shapes affective decision-making under uncertainty. Here, we focused on the important, yet unaddressed, question of how stress reactivity alters decisions about the valence of ambiguous social cues. Specifically, we causally manipulated stress exposure in half of our participants and measured how neuroendocrine responses to stress affect the perception of ambiguous facial expressions that could signal either positive or negative responses. Our stress manipulation was marked by significant increases in both subjective and neuroendocrine stress measures for the stress group only, confirming the efficacy of our manipulation. The considerable overlap between the neural circuitry that gives rise to valence bias and the large body of work suggesting that stress diminishes regulatory influence on reflexive affective processing collectively informed our hypothesis that this elevated stress response may bias individuals to view ambiguous faces with a higher degree of negativity. Indeed, we provide evidence that individual differences in stress reactivity—indexed by cortisol change relative to baseline—were associated with enhanced negative valence perception of ambiguous stimuli. Importantly, this relationship was selective to participants in the stress group, suggesting that stress-induced elevations in cortisol were required for this relationship to emerge, rather than basal levels alone. Although our stress manipulation did not yield group differences in valence bias, our findings are consistent with stress and decision-making research that shows individual differences in stress reactivity may be a useful index to identify vulnerabilities to the effects of stress on decision-making under uncertainty^28^.

These individual differences extended to an association between negativity ratings and the recovery of cortisol responses, albeit in the opposite direction. That is, steeper drops in cortisol during the stress recovery period were related to higher negativity ratings. Although we might have expected poorer cortisol recovery to be related to negative valence bias, clinical research has suggested that anxiety and trauma-related disorders can lead to blunted cortisol responses or oversensitive feedback responses (i.e., cortisol responses returning to baseline too quickly)^30^. It is therefore possible that rapid cortisol recovery seen in those with higher negativity valence bias suggests a latent vulnerability for anxiety or higher initial stress reactivity. However, it is equally plausible that participants with higher cortisol increase—and thus the stronger negativity bias—simply had more opportunity for cortisol to decline.

Using mouse-tracking technology, we were able to uniquely identify features of this decision process by continuously measuring how response trajectories evolved in real-time. In line with growing evidence that surprised faces are initially predisposed toward negative appraisals^8–11^, we found that those participants who arrived at a final decision of positivity showed response trajectories that indicated greater attraction toward the competing (negative) valence option than when arriving at a final decision of negativity. In other words, there was a much weaker attraction toward positive ratings for participants that ultimately assigned a negative valence to surprised faces. Additionally, higher cortisol reactivity as well as greater subjectively perceived stress from the CP task were associated with more direct trajectories to negative valence responses, suggesting that higher stress reactivity further facilitated decisions toward negative responses and possibly enhanced this initial negativity bias. Importantly, we did not see this relationship emerge between cortisol and surprised faces ultimately rated as positive, suggesting that rather than simply reducing noise in the decision process, stress appears to selectively facilitate arriving at a negative valence decision for ambiguous facial stimuli.

These data align with a growing body of work that shows that acute stress exposure diminishes regulatory influence on negative affective stimuli, including emotional faces^31^, negative images^32,33^, and other aversive stimuli^18,34^. Although our task did not explicitly instruct participants to adopt a regulatory strategy, ample research suggests that the initial negativity bias highlighted in past work may give rise to positive valence judgments with longer reaction times^11^, prefrontal involvement^5^, and individuals with greater neural development (i.e., adolescents vs. children)^35^. Therefore, one interpretation of our findings is that greater stress reactivity may diminish this regulatory influence or impair the subsequent higher-order processing needed to eventually arrive at subjective ratings of positivity. This notion is further supported by our self-report data, which revealed that higher stress reactivity and negativity ratings were both related to greater difficulties using goal-directed behavior and emotion-regulation. Collectively, our data point to heightened stress reactivity as conferring a propensity to selectively perceive ambiguous cues as negative. This is especially important given that subtle shifts in valence bias when evaluating ambiguous affective stimuli can shape subsequent emotional and behavioral responses.

This interpretation is consistent with what we know about the brain regions underlying negative valence bias and acute stress responses, namely the amygdala, which plays a central role in both processes^2,5^. The high receptor density for glucocorticoids in the amygdala makes it especially sensitive to cortisol release after stress exposure, thus this region is ideally positioned to bias appraisals of ambiguous stimuli toward a negative or threatening valence^20^. Additionally, emerging work suggests that early noradrenergic responses—shown previously to enhance amygdala response to fear faces^36^—can work synergistically with these slower-released glucocorticoid responses to further enhance amygdala activity even after a stressor has terminated^19,37^. Thus, we speculate that this enhanced amygdala activity, paired with a more global shift in neural processing toward threat detection^21^ and disrupted functioning of prefrontal circuits known to contribute to positive appraisals of these ambiguous social cues^5^, may likely contribute to the behavioral results seen here.

Finally, our results are consistent with research that suggests higher trait anxiety or traumatic exposure to stress conveys greater sensitivity to threat perception in uncertain contexts^38^. Relatedly, other work has shown that individuals with higher trait anxiety have a greater tendency to rate ambiguous expressions as fearful^39^, a finding that has also been found to alter representation of the amygdala as a function of anxiety^40^. More recently, a study assessing the effects of threat-of-shock on valence bias found that increased arousal motivated a shift toward more negative ratings for ambiguous facial expressions^41^. Here, we extend this research to healthy individuals and identify a causal role for acute stress responses in modulating these decision biases. Future research characterizing how acute stress and its concomitant neuroendocrine response alters the brain circuits during such affective decisions will enable researchers to test whether these effects are, in fact, driven by regionally-specific stress effects on amygdala-vmPFC circuitry. We also note that, unlike many economic decision-making tasks, our current design tested affective decisions without any decision outcomes, preventing feedback processing or learning mechanisms from playing a role in the valence bias seen here under stress. Therefore, our findings provide a first step to acquiring a more comprehensive understanding of how stress reactivity, such as that experienced in daily life, may drive affective appraisals and decisions that further shape important forms of behavior.
